# Left atrial strain improves estimation of filling pressures in heart failure: a simultaneous echocardiographic and invasive haemodynamic study

**DOI:** 10.1007/s00392-018-1399-8

**Published:** 2018-12-10

**Authors:** Anders Lundberg, Jonas Johnson, Camilla Hage, Magnus Bäck, Bela Merkely, Ashwin Venkateshvaran, Lars H. Lund, Anikó Ilona Nagy, Aristomenis Manouras

**Affiliations:** 1 Institution for Medicine, Solna, Karolinska Institutet, Stockholm, Sweden; 2 Department of Obstetrics and Gynecology, Centre for Fetal Medicine, Karolinska University Hospital, Stockholm, Sweden; 3 Theme of Heart and Vessels, Karolinska University Hospital, Stockholm, Sweden; 4 Heart and Vascular Center, Semmelweis University, Budapest, Hungary

**Keywords:** Left atrial strain, Non-invasive, Invasive, Exercise, Diastolic pressures

## Abstract

**Aims:**

Left ventricular diastolic pressure estimation is essential for characterization of heart failure (HF). Patients with normal resting left atrial (LA) pressures (LAP), but steep LAP elevation on exertion, pose a particular diagnostic challenge. Current recommendations on echocardiographic LAP estimation have limited accuracy. Our aim was to investigate whether LA mechanical alterations assessed by LA strain (LA-GS) can contribute to non-invasive LAP diagnostics.

**Methods and results:**

Simultaneous echocardiographic and right heart catheterization (RHC) data at rest and during exercise was analyzed in 164 prospectively enrolled patients, referred for RHC due to HF symptoms. 56% had preserved ejection fraction (pEF). At rest, 97 patients displayed elevated mean pulmonary arterial wedge pressure (PAWP_M_); further 32 patients had normal resting, but elevated PAWP_M_ during exercise. LA-GS demonstrated a stronger relationship with resting PAWP_M_ (*r* = − 0.61, *p* < 0.001) than any of the indices (*E*/*e*′, LAVi, TRV_max_) incorporated in the currently recommended diagnostic algorithm. The diagnostic ability of LA-GS for detecting elevated resting PAWP_M_ (AUC: 0.80, *p* < 0.001) outperformed that of the recommended algorithm (AUC: 0.69). Importantly, resting LA-GS performed even better in identifying patients with pathological PAWP_M_ either at rest or during stress (AUC: 0.90, *p* < 0.001), whereas the diagnostic potential of the current algorithm was modest and limited to pEF patients (AUC = 0.72). Finally, among the non-invasive indices, LA-GS entailed the strongest prognostic value for death or heart transplantation (OR: 2.7; *p* < 0.05).

**Conclusion:**

LA-GS comprises a robust method for PAWP_M_ assessment at rest. More importantly, it reliably discerns pathological PAWP_M_ rise on exertion despite normal resting pressures.

**Electronic supplementary material:**

The online version of this article (10.1007/s00392-018-1399-8) contains supplementary material, which is available to authorized users.

## Introduction

Diagnostic, prognostic and therapeutic reasons confer to the non-invasive estimation of left ventricular (LV) filling pressures fundamental importance when evaluating patients presenting with unexplained dyspnea as well as those with documented heart failure (HF). Despite extensive research and the availability of numerous metrics, the assessment of resting LV diastolic pressures is challenging. Even more puzzling is the non-invasive diagnostics of patients whose resting left atrial (LA) pressure (LAP) is within normal range, but increases abnormally steeply on exertion [1, 2].

Current guidelines recommend the use of a multi-parametric algorithm (ASE/EACVI algorithm) for LAP estimation [3]. However, despite significant improvements in feasibility [4], as compared to previous recommendations, validating studies have demonstrated that the proposed approach entails only moderate diagnostic value for resting LAP [5, 6], which is further restricted for identifying patients with excessive LAP elevation on exertion despite normal resting pressures [2]. Finally, although ventricular pacing is frequent in HF patients, there is ambiguity regarding the proper use of the currently recommended method in this cohort.

LA function has gained recognition as an important focus area in the assessment of LV diastolic function [7–9]. LA reservoir function is influenced both by the LV performance and by the intrinsic LA compliance and plays an important role in disease progression in various clinical states including HF [10–13]. Quantitative LA deformation analysis using speckle tracking has evolved as a highly feasible and reproducible method for evaluating LA function. It has been proven that LA global strain (LA-GS) is associated with mechanical changes and fibrosis even prior to LA geometrical remodeling [8, 14, 15], potentially enabling earlier diagnosis of elevated LAP. Previous studies have shown an inverse relationship between LA-GS and the diastolic pressures [5, 16]. Accordingly, we hypothesized that LA-GS might contribute to improve the non-invasive assessments of elevated LAP, and set out to assess its diagnostic value in that setting. Furthermore, we opted to investigate the applicability of LA-GS in patients on ventricular pacing.

## Methods

### Study population

All consecutive patients referred right heart catheterization (RHC) at the Karolinska University Hospital for the HF assessment between February 2014 and June 2017 (*n* = 220) were screened for enrollment. In all cases medical treatment had been titrated and hemodynamic stabilization achieved at the time of examination. No patient presented with acute coronary syndrome or had undergone cardiac surgery for a period of < 1 year prior enrollment. The study conformed to the Declaration of Helsinki, had ethics approval by local ethics committees and all participants provided written informed consent.

### Echocardiography

**Fig. 1:**
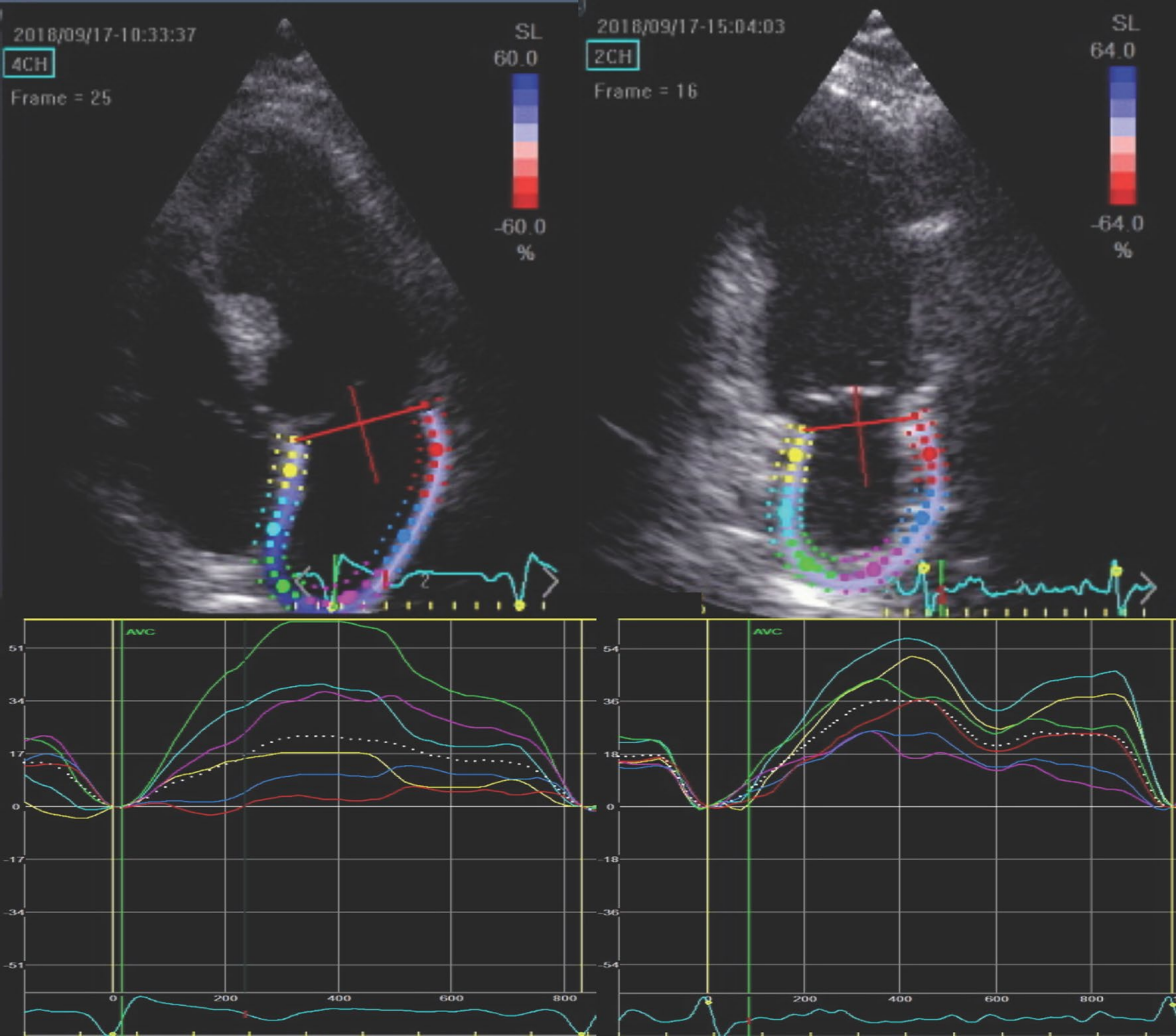
Left atrial strain curves obtained from four- and two-chamber views. The white dotted line indicates the mean strain value over the heart cycle in each view. LA reservoir function was estimated by peak LA longitudinal strain during ventricular systole. Global left atrial strain (LA-GS) was calculated by averaging the peak LA strain values from all 12 segments

All subjects underwent transthoracic echocardiography using an E9 system (GE Ultrasound, Horten, Norway) equipped with a 2.5 MHz matrix array transducer in keeping with current guidelines. 2D gray-scale images were acquired over 3 heart cycles and analyzed off-line (EchoPAC PC, version 11.0.0.0 GE Ultrasound, Waukesha, Wisconsin) by a single sonographer blinded to clinical and RHC data. LV end-diastolic, end-systolic volume and ejection fraction (EF) were measured using the Simpsons biplane method [17]. For LA volumetric analysis, the ‘method of disk’ method was employed. Myocardial deformation was analyzed by 2-D speckle tracking. LV global longitudinal strain (LV-GLS) was calculated as the average value of 12 segments obtained from the apical 4- and 2-chamber views. LA global strain (LA-GS) was assessed using images obtained in apical 4- and 2-chamber views (frame rate: 60–80 Hz), with attention for optimal visualization of the LA. Acoustic tracking was performed by semi-automated analysis. The endocardial border of the LA was traced manually so that the LA appendage and pulmonary veins were excluded, then an additional epicardial line was automatically generated by the software creating a region of interest (ROI), the shape of which was manually adjusted to precisely draw out the atrial contour and cover the full thickness of atrial myocardium (Fig. 1). Automatic processing was controlled by 1, visual confirmation of correct tracking of the endocardial border throughout the cardiac cycle and 2, by automated self-check function of the software. In case the tracking did not seem appropriate according to the reader or a segment was not approved by the software, repeated manual adjustment of the ROI was performed. If repeated processing did not result in an approved strain curve, the loop was excluded from the analysis (*n* = 4 cases). Following approval, the software automatically divided the LA endocardium into six segments, and longitudinal strain curves for each segment were generated. Zero point was set at the onset of the QRS complex on the ECG. LA reservoir function was estimated by peak LA longitudinal strain during ventricular systole. LA-GS was calculated by averaging strain measurements from all the segments. In patients in sinus rhythm, measurements from 3, in patients in AF (*n* = 42) measurements from 5 consecutive beats were averaged. Loops were carefully recorded so that PVCs/PACs were avoided. Reproducibility was tested in 40 randomly selected patients. The early (E) and late (A) mitral flow velocities were recorded using a 5 mm pulsed wave (PW) sample volume. Tricuspid regurgitation peak velocity (TRV_max_) was recorded employing continuous wave Doppler. Spectral tissue velocities were recorded in the septal and lateral mitral annulus using a 5 mm PW sample volume and the early myocardial relaxation velocity (*e*′) was recorded. The *E*/*e*′ ratio was calculated from the average of septal and lateral myocardial velocities. At the time of examination 42 patients were in AF.

### Catheterization

Within 1 h following echocardiography, RHC was performed using a 6F Swan-Ganz catheter through the jugular or femoral vein. Mean right atrial pressure, the systolic-, diastolic-, mean pulmonary artery pressure (PAP_M_) and the mean pulmonary artery wedge pressure (PAWP_M_) were recorded under fluoroscopy after calibration with the zero-level set at the mid-thoracic line. Pressure tracings were stored (WITT Series III, Witt Biomedical Corp., Melbourne, FL) and analysed offline. PAWP_M_ measurements were averaged from a minimum of 5 heart cycles at spontaneous end-expiration.

### Exercise protocol

Following the assessment of resting hemodynamics, patients with normal PAWP_M_ at rest (≤ 15 mmHg) performed supine cycle ergometry. Patients cycled at 60 rpm starting at a 20 W workload and increasing by 10 W increments in 1-min stages to maximum tolerated levels. PAWP_M_ was determined at peak exercise. Prior studies in normal controls have shown that peak PAWP_M_ during supine exercise are < 20–23 mmHg [18, 19]. In our study, PAWP_M_ ≥ 23 mmHg during peak exercise denoted abnormal LAP response.

### Biomarkers

NT-proBNP was analyzed by proBNPII (Roche Diagnostics, Bromma, Sweden). Estimated glomerular filtration rate (eGFR) was calculated according to the MDRD study equation: eGFR = 175 × [creatinine] − 1.154 × 10^6^−0.203 × 0.742 [if female] mL/min/1.73; Creatinine in mg/dL, age in years.

### Statistical analysis

The IBM SPSS statistics version 23.0 was used. QQ plots tested normality. Continuous variables are expressed as median and interquartile range. Categorical variables were expressed as absolute values and percentage. For comparisons of different groups, the Mann–Whitney test was used. Correlations were tested by the Pearson’s 2-tailed test or Spearman test, as appropriate. All tests were performed at 95% confidence intervals. A *p* value of < 0.05 was considered statistically significant.

The association of invasive and non-invasive diastolic indices with the combined outcome of death or heart-transplantation (HTX) was tested using a time to event analysis with univariate and multivariable Cox proportional hazards models, adjusting for demographic, clinical and echocardiographic covariates (*E*/*e*′, TRV_max_, age, hypertension, diabetes, LV-EF, ln[NT-proBNP] and eGFR) and a Kaplan–Meier non-parametric test and compared employing a log-rank test. The proportional hazards assumption was tested for all analyses. Receiver-operating characteristic (ROC) curves were plotted for individual parameters regarding the discrimination of increased PAWP_M_ and to establish the optimal cut-off points.

NT-proBNP data were natural logarithmically transformed. Analysis of intra-observer variability was performed for LA-GS by double measurements in 40 randomly selected patients. Inter-observer variability together with test–retest reliability was tested in 29 individuals, who have undergone two consecutive examinations each, within 20 min, performed by two different examiners and then the two recordings analysed again by two different readers.

## Results

### Patient characteristics

Demographic data are provided in Table 1. In total 220 patients referred for diagnostic right heart catheterization (RHC) with suspected HF or unexplained dyspnea were prospectively screened. Of the 220 patients catheterized, 11 patients who previously underwent cardiac transplantation with atrial anastomosis technique were excluded. By the results of catheterization, 29 patients turned out to have an underlying disease other than primary left heart myocardial disease (isolated pre-capillary arterial pulmonary hypertension, *n* = 15; constrictive pericarditis, *n* = 14), and therefore, were excluded from further analysis. In addition, 3 patients with ARVD, 10 patients with significant valvular disease and 3 patients with inadequate echocardiographic image quality were also excluded from further analysis (Figure S1).

**Table 1:** Demographic data stratified according to EF groups

General characteristics	EF ≥ 50%	EF < 50%	*p* value
	(*n* = 92)	(*n* = 72)	
Age years	73 (67; 79)	58 (47; 64)	< 0.001
Female	57 (62%)	15 (21%)	< 0.001
**Medical history and symptoms **			
AF	44 (48%)	33 (46%)	0.799
PM/CRT	8/0 (9%)	8/16 (33%)	
Hypertension	63 (69%)	42 (58%)	0.30
Diabetes mellitus	17 (19%)	13 (18%)	0.84
Hypercholesterolaemia	22 (24%)	23 (32%)	0.197
Ischaemic heart disease	3 (3%)	19 (26%)	< 0.001
NYHA I	12 (13%)	1 (1%)	
NYHA II	18 (20%)	9 (13%)	
NYHA III	59 (64%)	56 (78%)	
NYHA IV	3 (3%)	6 (8%)	
**Clinical measurements **			
BMI kg/m2	27 (23; 30)	27 (24; 31)	0.63
Obesity (BMI ≥ 30)	25 (27%)	19 (26%)	0.911
SBP (mm Hg)	130 (117; 142)	101 (93; 115)	< 0.001
DBP (mm Hg)	67 (60; 71)	62 (55; 70)	0.04
HR (beats/min)	70 (60; 80)	68 (59; 77)	0.51
*Treatment*			
ARB or ACE-I	50 (54)	64 (89)	< 0.001
Loop diuretic	63 (69)	65 (90)	0.001
Beta blocker	61 (66)	68 (94)	< 0.001
MRA	30 (33%)	53 (74%)	< 0.001
Calcium channel blocker	26 (28)	5 (7)	= 0.001
**Laboratory findings **			
NT-proBNP (ng/L)	1250 (365; 2300)	2580 (1265; 2693)	< 0.001
eGFR (mL/min/1.73 m^2^)	65 (46; 88)	81 (68; 102)	< 0.001
Hemoglobin (mg/L)	123 (113; 140)	138 (125; 148)	< 0.001

Categorical variables are provided as absolute numbers followed by percentages in brackets; continuous variables or median values followed by 1st and 3rd quartiles in brackets

*EF* ejection fraction, *AF* atrial fibrillation or flutter, PM pacemaker, *CRT* cardiac resynchonization therapy, *NYHA* New York Heart Association functional class, *BMI* body mass index, *SBP* systolic blood pressure, *DBP* diastolic blood pressure, *HR* heart rate, *ARB* angiotensin receptor blocker, *ACE-I* ACE-inhibitor, *MRA* mineralocorticoid receptor antagonist, *NT-proBNP* N-terminal pro-brain natriuretic peptide, *eGFR* estimated glomerular filtration rate

In effect, recordings of 164 patients were analyzed (age 63 ± 15; 74 female). Median EF was 54% (Q1:25%, Q3:62%), 72 patients (44%) had EF < 50% (HFrEF). At the time of enrollment all patients were symptomatic. Ischemic cardiomyopathy was the cause of HF in 26 cases, idiopathic dilated cardiomyopathy in 53, restrictive cardiomyopathy of various origin in 23 (amyloidosis: 5, sarcoidosis: 1, hypertrophic: 5, other: 12), and viral myocarditis in 1 case, with the rest being of multifactorial origin.

At rest, 97 patients (59%) demonstrated elevated PAWP_M_ (> 15 mmHg). Additionally, 32 patients with normal resting PAWP_M_ displayed abnormal LAP (≥ 23 mmHg) during exercise [preserved EF (pEF), *n* = 17 (19%); HFrEF, *n* = 15 (21%)]. In total 129 (79%) patients had elevated LAP either at rest or during exertion. In 8 cases, the exercise testing was not feasible due lower extremity pain (*n* = 3) or inability to follow instructions (*n* = 5). Echocardiographic and invasive measurements for pEF and HFrEF are summarized in Table 2.

**Table 2:** Cardiac geometric and functional measures in the two groups stratified according to EF

	EF ≥ 50% (*n* = 92)	EF < 50% (*n* = 72)	*p* value
**LV dimensions **			
LV EDVi (mL/m^2^)	47 (39; 61) (88)	99 (78; 132) (62)	< 0.001
LV ESVi (mL/m^2^)	16 (12; 24) (88)	75 (46;101) (62)	< 0.001
LVMi (g/m^2^)	88 (69; 111) (88)	139 (103; 164) (71)	< 0.001
*LV systolic function*			
LV-EF (%)	60 (57; 65) (92)	25 (20; 40) (72)	< 0.001
LV-GLS (%)	18 (14; 20) (91)	6.8 (4.9; 10) (71)	< 0.001
**LV diastolic function**			
E/A ratio	1.3 (0.9; 2.1) (60)	2.9 (1.7; 3.9) (57)	< 0.001
*e*′ septal	6.9 (5.0; 8.0) (92)	4.5 (3.3; 60) (72)	< 0.001
*e*′ lateral	8.1 (6.6; 10.9) (92)	7.5 (6.0; 10.0) (72)	NS
*E*/*e*′	12.5 (9.5; 18.4) (91)	15.3 (11.5; 23.7) (72)	0.015
**LA function**			
LA ESVi (mL/m^2^)	42 (34; 53) (92)	60 (44; 69) (72)	< 0.001
LA-GS (%)	12.0 (7.0; 17.9) (90)	7.9 (5.0; 10.1) (70)	< 0.001
**RV parameters**			
TAPSE (mm)	17 (13; 22) (92)	14 (11; 17) (71)	0.002
RVSP (mmHg)	44 (37; 58) (90)	46 (36; 57) (66)	NS
**Hemodynamic**			
PAWP_M_ (mmHg)	16 (13; 22) (91)	19 (14; 25) (72)	0.023
PAP_M_ (mmHg)	26 (21; 37) (92)	29 (22; 37) (72)	NS
Cardiac Index (mL/m2)	2.5 (2.0; 2.9) (92)	2.0 (1.6; 2.3) (72)	< 0.001

Data are provided as median values followed by 1st and 3rd quartiles in brackets. Patient numbers for each measurement are provided in brackets

*BMI* body mass index, *HR* heart rate, *SBP* systolic blood pressure, *DBP* diastolic blood pressure, *LV* left ventricle, *EDVi* end-diastolic volume index, *ESVi* end-systolic volume index, *LVMi* LV mass index, *EF* ejection fraction, *LV-GLS* LV global longitudinal strain, *E/A* ratio between the early diastolic inflow velocity (*E*) to the inflow velocity due to atrial contraction (A), *e*′ mean, mean value of early myocardial velocity in LV basal septal and lateral wall, *E*/*e*′ ratio between the *E* and the *e*′, *LA* left atrium, *LA ESVi* left atrial end-systolic volume, *LA-GS* left atrial global longitudinal strain, *TAPSE* tricuspid annular plane systolic excursion, *RVSP* right ventricular systolic pressure as assessed by echocardiography, *PAWP*_*M*_ pulmonary arterial wedge pressure, *PAP*_*M*_ pulmonary arterial mean pressure, *NS* non-significant (*p* ≥ 0.05)

At the time of examination, 42 (26%) patients were in atrial fibrillation (AF) and 32 patients (19%) had continuous ventricular pacing (VP) (CRT in 16 cases).

### Echocardiographic metrics and PAWP

In the whole cohort, PAWP_M_ showed a significant inverse correlation with LA-GS (*r* = − 0.54, *p* < 0.001) and weaker albeit significant relationship with TRV_max_, LAVi and *E*/*e*′ (*r* = 0.41, 0.29, 0.28, respectively; *p* < 0.001 for all). The ASE/EACVI algorithm for diastolic assessment does not apply to patients in AF or those with ventricular pacing. Nonetheless, as LA-GS has not yet been thoroughly investigated in this setting, we decided to study its validity in these specific subgroups. Conceivably, AF might importantly influence LA-GS measurements. Accordingly, separate analysis of the AF group confirmed that neither LA-GS (*r* = − 0.26, *p* = 0.092), nor LAVi (*r* = − 0.116, *p* = 0.46) or *E*/*e*′ (*r* = 0.21, *p* = 0.19) were associated with PAWP_M_. In contrast, in the VP group, PAWP_M_ significantly correlated with LA-GS (*r* = − 0.54, *p* = 0.002) and *E*/*e*′ (*r* = 0.41 *p* = 0.021), but lacked association with either LAVi or TRV_max_ (*r* = − 0.14 and 0.2, *p* > 0.05 in both cases).

Confining the analysis to patients in sinus rhythm (SR) (i.e., where the currently recommended algorithm is applicable), the correlations between PAWP_M_ and LA-GS, LAVi and TRV_max_ were stronger (*r* = − 0.66, 0.51 and 0.46 respectively, *p* < 0.001 for all). However, the *E*/*e*′ remained only weakly associated with PAWP_M_ (*r* = 0.25, *p* < 0.018). Accordingly, we decided to proceed with all further analysis in the cohort in regular rhythm (RR), i.e., both those in SR and VP and to exclude patients with AF.

In a multivariable analysis including LA-GS, LAVi, TRV_max_, *E*/*e*′ and LV-GLS, the LA-GS was identified as the strongest predictor of PAWP_M_ with a partial correlation of *r* = − 0.42 (*p* < 0.001) in RR patients. The correlation for TRV_max_ was *r* = 0.33 (*p* < 0.001), while the other variables were not associated with PAWP_M_ (*p* > 0.05). Similarly, in the SR cohort, LA-GS and TRV_max_ were identified as the only predictors of PAWP_M_ (partial correlations *r* = − 0.40 and *r* = 0.33 respectively, *p* < 0.001), whereas the other variables were not associated with PAWP_M_ (*p* > 0.05). No correlation between LV-GLS and PAWP_M_ was found in either EF group (*p* > 0.05).

**Fig. 2:**
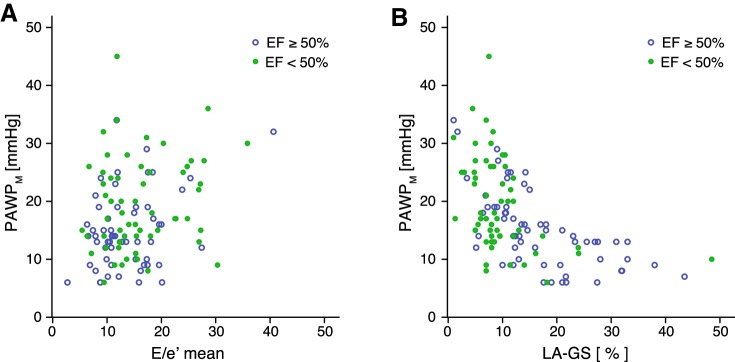
Correlation of invasively measured PAWP_M_ values with left atrial global strain (LA-GS) (**a**) and *E*/*e*′ (**b**) in patients with regular rhythm with preserved (HFpEF) or reduced (HFrEF) ejection fraction

As shown in Table 3a, when dividing our RR cohort based on the EF, compared to the other non-invasive metrics, LA-GS displayed the strongest correlation with PAWP_M_ in both EF groups (*r* = − 0.61 and − 0.46 in pEF and HFrEF, respectively, *p* < 0.001 for both) (Fig. 2). Notably, the correlation between LA-GS and PAWP_M_ was strongest in the preserved EF group; further stratification of our cohort into moderately and severely reduced EF confirmed a progressively weaker LA-GS–PAWP_M_ correlation along with lower  EF ranges (EF 30–49%: *r* = − 0.54 *p* = 0.014; EF < 30%, *r* = − 0.36 *p* = 0.03). Echocardiographic and haemodynamic characteristics of these subgroups are provided in Table S1. LAVi and TRV_max_ were also significantly related to PAWP_M_ (*p* < 0.05), whereas the correlation of *E*/*e*′ with PAWP_M_ was confined to pEF patients (*r* = 0.32, *p* = 0.002).

**Table 3:** Sensitivity, specificity and area under curve values of various echocardiographic estimates for identifying elevated LAP in patients with preserved or reduced EF (A); at rest and during stress, independent of the EF (B)

A		EF ≥ 50%						EF < 50%					
		*r*	*p*	SP	SN	AUC	*p*	*r*	*p*	SP	SN	AUC	*p*
**Regular rhythm**		(*n* = 63)						(*n* = 59)					
Rest	*E*/*e*′	0.33	0.012	67	62	0.68	0.016	0.17	0.20			0.59	0.26
	LAVi	0.39	0.002	53	96	0.79	< 0.001	0.29	0.025	21	97	0.66	0.041
	TRV_max_	0.55	< 0.001	56	71	0.68	0.019	0.37	0.006	58	82	0.70	0.018
	LA-GS	− 0.64	< 0.001	77	93	0.87	< 0.001	− 0.46	< 0.001	61	68	0.74	0.002
	ASE/EACVI			44	93	0.69	0.013			–	–	0.62	0.14
	Rest-Cath			100	70	0.85	< 0.001			100	70	0.85	0.002
Stress	*E*/*e*′	–	–			0.56	0.46	–	–			0.57	0.53
	LAVi	–	–	69	85	0.77	0.002	–	–	–	–	0.67	0.131
	TRV_max_	–	–			0.66	0.076	–	–			0.71	0.121
	LA-GS	–	–	88	92	0.93	< 0.001	–	–	77	96	0.85	0.003
	AS/EACVI			56	88	0.72	0.011					0.58	0.448
**Sinus rhythm**		(*n* = 55)						(*n* = 35)					
Rest	*E*/*e*′	0.35	0.014	65	61	0.66	0.042	0.10	0.551			0.60	0.340
	LAVi	0.43	0.001	61	96	0.81	< 0.001	0.48	0.004	31	96	0.74	0.022
	TRV_max_	0.55	< 0.001	21	74	0.70	0.015	0.38	0.042			0.69	0.120
	LA-GS	− 0.66	< 0.001	78	86	0.90	< 0.001	− 0.55	0.001	59	86	0.78	0.008
	ASE/EACVI			48	91	0.70	0.013			33	96	0.64	0.042
	Rest-Cath			100	72	0.86	< 0.001			100	73	0.87	0.019
Stress	*E*/*e*′					0.58	0.38					0.59	0.58
	LAVi			69	91	0.75	0.005			–	–	0.72	0.165
	TRV_max_					0.57	0.439					0.58	0.699
	LA-GS			88	90	0.92	< 0.001			100	97	0.98	0.003
	ASE/EACVI			56	84	0.70	0.023			–		0.57	0.64

B		Rest						Stress			
		*r*	*p*	SP	SN	AUC	*p*	SP	SN	AUC	*p*
**Regular rhythm** **(** ***n*** ** = 122)**											
Rest-Cath								100	70	0.85	< 0.001
*E*/*e*′		0.28	0.002	63	57	0.65	0.005			0.57	0.307
LAVi		0.40	< 0.001	40	97	0.75	< 0.001	58	91	0.75	< 0.001
TRV_max_		0.40	< 0.001	61^a^	65^a^	0.65	0.002	–	–	0.55	0.400
LA-GS		− 0.61	< 0.001	70	80	0.80	< 0.001	83	89	0.90	< 0.001
ASE/EACVI				31	94	0.66	0.003	46	90	0.68	0.007
**Sinus rhythm** **(** ***n*** ** = 90)**											
Rest-Cath								100	73	0.86	< 0.001
*E*/*e*′		0.25	0.018	66	53	0.64	0.023			0.57	0.377
LAVi		0.51	< 0.001	52	96	0.80	< 0.001	65	87	0.76	< 0.001
TRV_max_		0.46	< 0.001	24	75	0.69	0.004	–	–	0.50	0.95
LA-GS		− 0.63	< 0.001	64	96	0.86	< 0.001	84	93	0.94	< 0.001
ASE/EACVI				38	93	0.69	0.002	50	89	0.68	0.014

Data are provided separately for patients in regular rhythm (patients with atrial fibrillation excluded) and those in sinus rhythm (patients with atrial fibrillation and/or pacemaker rhythm excluded). Patient numbers for each cohort are provided in brackets

*r* correlation coefficient, *AUC* area under curve provided by ROC analysis, *SP* specificity, *SN* sensitivity, *LAVi* left atrial volume index, *TR-V*_*max*_ maximal velocity of the tricuspid regurgitant jet, *LA-GS* left atrial global strain, followed by the applied cut-off values for pEF and rEF, respectively in brackets, *ASE/EACVI* the algorithm recommended by the current guidelines, *rest-cath* resting PAWP_M_ value obtained by invasive measurement, *EF* ejection fraction

^a^Using a cut off value of 2.9 m/s

### Correlation between non-invasive markers and NT-proBNP

In patients with RR, NT-proBNP was strongly associated with LA-GS (*r* = − 0.64, *p* < 0.001) with significant but weaker correlation with PAWP_M_, LAVi (*r* = 0.40 and 0.52, respectively; *p* < 0.001 for both) and *E*/*e*′ (*r* = 0.25; *p* = 0.012) but not with TRV_max_ (*p* > 0.05). In a multivariable analysis including LA-GS, LAVi, TRV_max_, *E*/*e*′ and LV-GLS, the LA-GS remained the sole predictor of NT-proBNP with a partial correlation of *r* = − 0.43 (*p* < 0.001).

### Echocardiographic metrics for elevated LAP at rest and during exercise

**Fig. 3:**
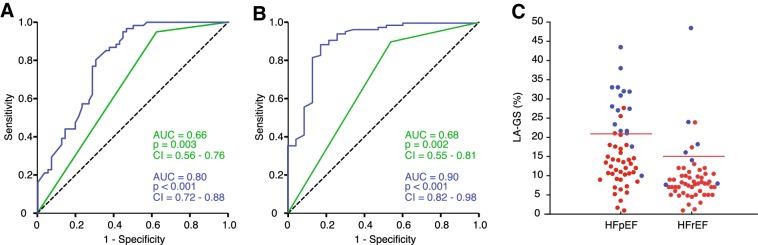
Diagnostic accuracy of LA-GS. ROC curve of LA-GS (blue) and the ASE/EACVI algorithm (green) for identifying elevated PAWP_M_ at rest (**a**) and at rest and/or during stress (**b**), independent of the EF. Beeswarm plot showing subjects with normal (blue) or pathological (red) PAWP_M_ values at rest and/or during exercise, dichotomized according to the EF (**c**). Optimal LA-GS cut-off values for preserved and reduced EF are 21% and 17%, respectively. LA-GS, left atrial global strain; ROC, receiver operating characteristics; PAWP_M_, mean pulmonary arterial wedge pressure; EF, ejection fraction

As illustrated in Table 3, LAVi and the ASE/EACVI algorithm provided fairly good ability to rule out elevated resting PAWP_M_. However, their specificity was limited and for the ASE/EACVI algorithm restricted to pEF patients. In contrast, LA-GS yielded a more robust discrimination for resting PAWP_M_, particularly in the pEF group (LA-GS cut-off: 15%, specificity and sensitivity 83% and 89%, respectively; *p* < 0.0001). HFrEF patients displayed generally lower LA-GS values, therefore, at an optimal cut-off value of 8% in this cohort, a specificity of 61% and sensitivity of 68% was achieved (*p* = 0.002) (Fig. 3A).

Even more diagnostically challenging are those patients displaying resting LAP within the normal range, but an abnormal pressure elevation on exertion. Importantly, we found that taking into account all patients with elevated LAP either at rest or during exercise, the predictive value LA-GS improved further. In patients with pEF, resting LA-GS (cut-off: 21%) displayed a specificity and sensitivity of 88% and 92%, while in HFrEF (cut-off 15%) the corresponding values for identifying pathological LAP at rest or during stress were 67% and 92%, respectively (Fig. 3c). Noticeably, invasive PAWP_M_ measurements at rest had lower sensitivity compared to LA-GS in this diagnostic setting. Finally, the ASE/EACVI algorithm demonstrated limited specificity in pEF patients (specificity: 56%, sensitivity: 88%, AUC = 0.72; *p* = 0.011) and in fact no significant diagnostic information in the HFrEF group (Fig. 3b).

Recently, a multivariable approach based on echocardiographic (Doppler derived pulmonary artery systolic pressure, *E*/*e*′) and clinical (age, BMI, hypertension, atrial fibrillation) parameters for HFpEF diagnosis as assessed by invasive rest and exercise testing [20]. A weighted score based on the above 6 parameters (H_2_FPEF) provided robust discernment of HFpEF (AUC 0.84). In our cohort, the mean H_2_FPEF was 5 (3% of the patients had score 0; 5% score 1; 3% score 2; 15% score 3; 16% score 4; 8% score 5; 14% score 6; 23% score 7; 3% score 8 and 9% had score 9). ROC analysis of the predictive ability of the score in the subgroup of patients with EF > 50% was 0.81 (0.71–0.91); *p* < 0.001. H_2_FPEF score and the LA-GS demonstrated a significant inverse relationship (*r* = − 0.51, *p* < 0.001). The corresponding association between the H_2_FPEF score and the PAWP at rest was *r* = 0.44, *p* < 0.001, whereas there was no significant association with *E*/*e*′ (*r* = 0.12; *p* > 0.05).

### Indices of diastolic function and outcome

**Fig. 4:**
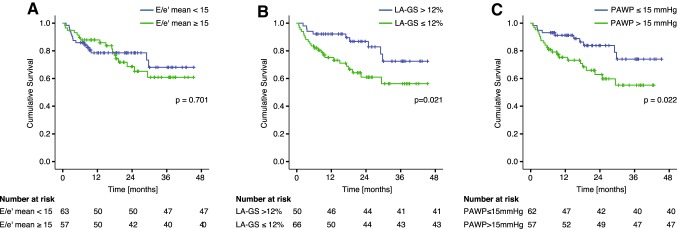
Prognostic value of LA-GS. Kaplan–Meier analysis of the predictive value of *E*/*e*′ (**a**), LA-GS (**b**) and invasively measured PAWP_M_ (**c**) on the composite outcome of death and/or HTX. LA-GS, left atrial global strain; PAWP_M_, mean pulmonary arterial wedge pressure; HTX, heart transplantation

The prognostic information provided by non-invasive and invasive metrics of LAP was subsequently evaluated. Using Kaplan–Meier analysis the predictive value of LA-GS (cut-off: < 12%), PAWP_M_ (cut-off: > 15 mmHg) and *E*/*e*′ (cut-off: > 14) was investigated (Fig. 4**)**. Over a median follow-up of 561 days (IQR: 270–839) 32 primary outcome events occurred [20 deaths, 12 HTX]. No patients were lost to follow-up. PAWP_M_ > 15 mmHg was related with higher risk for death or HTX [hazard ratio (HR) 2.3; confidence interval (CI) 1.1–4.9, *p* = 0.022]. Furthermore, LA-GS < 12% was associated with an increased risk for the primary composite endpoint in unadjusted analysis [HR: 2.4, CI 1.1–5.2, *p* = 0.029] and was the sole independent predictor of outcome when adjusted for *E*/*e*′ and TRV_max_, using the cut-off values recommended in the ASE/EACVI algorithm [HR: 2.8; CI 1.1–6.1, *p* = 0.017]. However, when adjusted for age, eGFR, hypertension, diabetes mellitus, LV EF and ln[NT-proBNP], neither the LA-GS nor the PAWP_M_ retained their predictive potential, NT-proBNP remaining the sole significant prognostic index (HR 1.53; *p* = 0.022) (Table S2).

### Feasibility and reproducibility

Measurements of LAVi and *E*/*e*′ could be performed in all cases whereas the feasibility of LA-GS and TRV_max_ were 98% and 95%, respectively. In the present study the ASE/EACVI algorithm provided a definite diagnosis in 99% of the cases, with only 1 case remaining indeterminate.

Double measurements for LA-GS in 40 randomly selected patients demonstrated a coefficient of variation of 10% with intra-class correlation coefficient 0.91 (95% CI 0.73–0.96).

Test retest analysis for the LA-GS yielded high reliability with a slightly higher coefficient of variation of 12.8%.

## Discussion

In this prospective study we demonstrate that in patients with known or suspected HF, LA-GS provides a feasible and robust diagnostic tool for the identification of elevated LAP either at rest or on exertion, with this single measurement entailing higher diagnostic accuracy as compared to the currently recommended diagnostic algorithm. Furthermore, we show that among the tested non-invasive indices of filling pressures, LA-GS provides the best prognostic value in HF patients.

### Recommended algorithm for the assessment of diastolic dysfunction

Given the limited individual accuracy of the established non-invasive metrics, the evaluation of LV filling pressures requires a number of parameters to be taken into account [21], rendering diastolic assessment a laborious task. In 2016 the American Association of Echocardiography (ASE) together with the European Association of Cardiovascular Imaging (EACVI) published a new joint guideline proposing a novel algorithm, based on expert consensus [3], that comprises a simplified thus potentially more feasible approach for echocardiographic LAP estimation. A number of studies have since tested the diagnostic validity of this new algorithm and concluded that it provides an improved yet still moderate sensitivity for elevated LAP [4–6, 21] along with fewer indeterminate cases compared to the previous recommendations [4]. Our findings essentially corroborate these observations as we show that despite good feasibility, the diagnostic potential of the ASE/EACVI algorithm remains modest. More specifically, as compared to previous findings [2, 6] we show that the ASE/EACVI algorithm provided better ability for ruling out elevated LAP, compromised, however, by a poorer specificity, probably due to the higher prevalence of severe HF in our cohort.

### LA-strain for the assessment of resting LAP

Normally, the distensible LA accommodates the inflowing volume from the pulmonary veins with only a slight rise in the LAP. However, in HF structural and pro-inflammatory alterations often result in increased LA-wall stiffness, shifting the LA pressure–volume curve upwards with accentuated pressure elevation for the same volume entering the LA [22]. It appears, therefore, pathophysiologically sound to postulate that characterization of LA-wall mechanics might contribute to the diagnostics of diastolic function. Indeed, LA wall deformation as quantified by LA-GS has been shown to correlate well with LV filling pressures in systolic HF [5, 7, 14, 23]. Moreover, Hummel et al. demonstrated that in HFpEF, as compared to established non-invasive metrics, LA-GS exhibited a stronger relationship with PAWP_M_ [5]. In line with these observations we show that in both HFpEF and HFrEF patients, the predictive potential of LA-GS for elevated resting LAP was superior not only to individual established non-invasive markers, but to the ASE/EACVI algorithm as well. Particularly in pEF patients, the fairly high specificity of LA-GS was accompanied by a comparable ability for ruling out elevated resting LAP.

### Diagnosis of patients with pathologically elevated resting or exercise-induced LAP

Notwithstanding the evident challenge in regard to non-invasive estimation of resting LAP, the diagnosis of patients presenting with exertional dyspnea comprises an even more perplexing undertaking. This becomes apparent in light of the findings of a recent study in which roughly 40% of HFpEF patients exhibited normal resting LAP, but displayed abnormal pressure elevation during exercise [5]. In our investigation, 20% of the invasively diagnosed HFpEF and HFrEF patients had normal resting, but elevated exercise LAP. Overall, the sensitivity of resting invasive hemodynamics for identifying abnormal diastolic response during exertion was higher yet still moderate (70%) as compared to the aforementioned study [2], a discrepancy that might be ascribed to the higher prevalence of more severe HF in our cohort as indicated by the higher NT-proBNP and LAVi values. Although the ASE/EACVI algorithm was not specifically developed for this purpose, given this being the currently recommended method for the diagnosis of diastolic dysfunction it is plausible to assume that patients with exercise induced pressure elevation and symptoms might score abnormal by this method. Obokata and colleagues were the first to validate the ASE/EACVI algorithm against the gold standard invasive exercise testing, demonstrating a fairly good specificity (80%) limited, however, by poor sensitivity (34%) in HFpEF [2]. Employing similar methodology our results show better sensitivity values and poorer specificity most possibly due to the more advanced HF in our study. Importantly, the ASE/EACVI approach applies to both patients with HFpEF and HFrEF [3]. However, according to the current results, in HFrEF the multi-parametric approach at rest was not predictive for abnormal LAP elevation during exercise.

In contrast, LA-GS had even further increased discriminatory capacity for pathological LAP when both resting- or exercise-induced values were taken into account. In fact, particularly in patients with pEF, resting LA-GS provided superior sensitivity than resting PAWP_M_ implying that LA-GS might be more sensitive in ruling out elevated PAWP_M_ during physical exertion than invasive examination in resting condition. LA-GS has been shown to inversely associate with the degree of LA-wall fibrosis as assessed by delayed-enhancement magnetic resonance imaging [24]. Furthermore, in patients undergoing mitral valve surgery, LA-GS comprised the strongest independent predictor of histologically quantified LA-wall fibrosis [14]. Conceivably, LA-GS might reflect the state of LA compliance, thus allowing identification of elevated LAP at rest but even more predicting abnormal LAP response at states of increased flow conditions such as during exercise.

The physiologic concept of LA-LV volume reciprocity describes the inherent association between the LV and LA function whereby the LV systolic deformation impacts on LA mechanics [7, 22]. Indeed, the current results substantiate this notion showing statistically significant association between LV-GLS and LA-GS (pEF: *r* = 0.36, *p* = 0.005; HFrEF: *r* = 0.38, *p* < 0.003). Nevertheless, as opposed to LA-GS, no correlation between LV-GLS and PAWP_M_ was found in either HF group (*p* > 0.05). More importantly, multivariate analysis demonstrated that LA-GS entailed an independent predictive value for LAP, whereas LV-GLS lacked diagnostic potential in that setting. This is not surprising as LA-GS quantifies mechanical events at the LA level associated with PAWP_M_, as opposed to LV-GLS, which might better reflect LVEDP. Additionally, previous experimental studies have demonstrated differential cellular responses with more pronounced pro-fibrotic changes detected in the LA as compared to the LV wall [25, 26] which advocates for LA-GS entailing a diagnostic value that is independent of the LV-GLS [15].

As the current results reveal, the specificity of LA-GS for resting and/or exercise LAP elevation was higher in patients with pEF which might be ascribed to the disparate degree of LA remodeling with smaller LAVi in pEF (pEF: 42 ± 15 vs. HFrEF: 55 ± 17 mL/m^2^, *p* < 0.001). Indeed, the association between LA-GS and PAWP_M_ was lower in patients with severely dilated LA (LAVi > 48 mL/m^2^) compared to those with mild to moderate LA enlargement (*r* = 0.69 vs. 0.30, *p* < 0.001). Although LA-GS is angle independent, extensive geometrical changes in LA might impact on the myocardial tracking. Additionally, the attenuation of wall stress secondary to LA dilatation might affect the reliability of LA-GS in predicting LAP [27].

### Outcome

Filling pressures not solely impact on symptoms, but on prognosis as well as previously shown [1]. Employing the currently recommended PAWP_M_ value (> 15 mmHg) demonstrated an increased event rate (death or HTX) in the elevated LAP group. However, when the non-invasive metrics of LAP were tested at the suggested discriminating values, only LA-GS comprised a prognostic value. Following adjustment for demographic and clinical parameters, neither the PAWP_M_ nor LA-GS remained diagnostic. The current findings might reflect inadequate power, but advocate as well for the complexity of the HF as a clinical entity.

### Clinical utility

In our study, LA-GS alone provided higher diagnostic accuracy for resting or exercise-induced pathological LAP elevation in HF patients, as compared to the multi-parametric ASE/EACVI algorithm. Thus, routine LA-GS measurements might provide a simple and robust tool in the setting of HF diagnostics and prognostication. Nevertheless, despite LA-GS constituting the best single non-invasive metric, its correlation with invasively measured pressures was still only moderate. Especially in case of reduced LA-GS, there was a considerable overlap between patients with normal and elevated LAP values. This ultimately advocates for the preferential use of various metrics instead of single measurements in the setting of LAP estimation. In addition, future studies are warranted for evaluating the possible incremental values of LA-GS measurements in context of novel HF device therapies, such as the inter-atrial shunt device implantation [28–30].

### Limitations

This is a single center study performed on a limited number of patients, still to the best of our knowledge it is the largest of its kind with echocardiographic and parallel resting and stress invasive measurements reported. Referral for RHC constitutes an important selection bias;, therefore, whether LA-GS is a better discriminator of elevated LAP compared to other non-invasive indices in a non-selected population cannot be assessed. The present study did not include a validation cohort, which warrants further studies to validate the established cut-off values as a necessary next step. AF is common in HF. Similarly, to other echocardiographic indices, according to our results LA-GS does not reliably reflect LAP in AF, which hinders the routine applicability of this metric. In contrast, LA-GS remained a reliable marker in patients on VP; however, the sample size of only 32 patients in this subgroup warrants confirmation in larger studies. Importantly, LA-GS measurements are not directly comparable to results obtained by deformation analysis packages from different vendors, thus the current results might not be representative in studies performed with different instrumentation. Finally, our cohort included a large number of highly symptomatic patients with extensive LA remodeling, warranting validation of this approach in less severe cases.

## Conclusion

We demonstrate that LA-GS comprises a feasible and fairly accurate tool for the identification of abnormally elevated LAP either at rest or on exertion in patients with known or suspected heart failure. This together with the revealed prognostic value in HF patients advocates for the incremental value of LA-GS in non-invasive HF diagnostics.

## Electronic supplementary material

Below is the link to the electronic supplementary material.

**Figure S1**. Patient selection process. Distribution of patients according to invasive pressure measurement results. RHC, right heart catheterization; HF, heart failure; PH, pulmonary hypertension; ARVC, arrhythmogenic right ventricular cardiomyopathy; PAWP_M_, mean pulmonary capillary wedge pressure as measured by RHC (PNG 40 KB)
