# Supplementary figures and images for: Left atrial strain improves estimation of filling pressures in heart failure: a simultaneous echocardiographic and invasive haemodynamic study
Source: Clin Res Cardiol. 2018 Dec 10;108(6):703–15. doi: 10.1007/s00392-018-1399-8 (PMC6529379; doi:10.1007/s00392-018-1399-8)

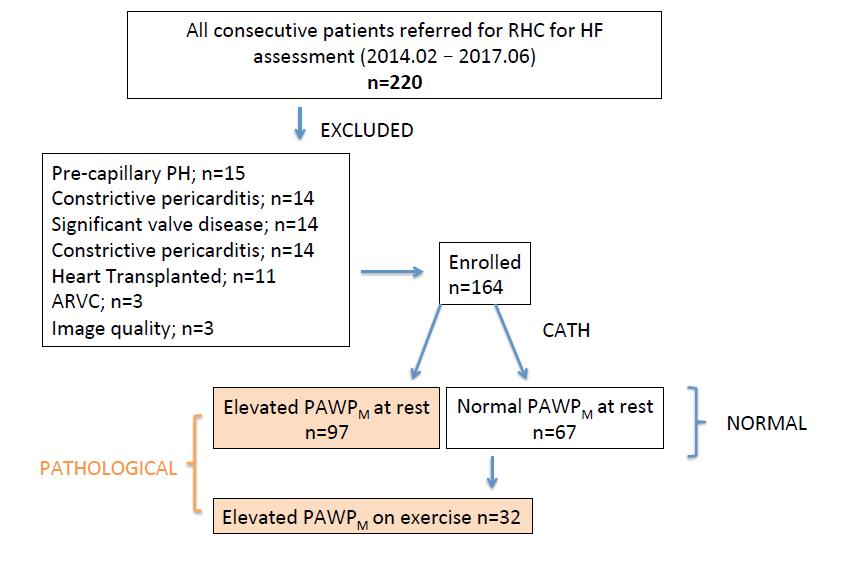

Supplement: Supplementary file 1 — Figure S1. Patient selection process. Distribution of patients according to invasive pressure measurement results. RHC, right heart catheterization; HF, heart failure; PH, pulmonary hypertension; ARVC, arrhythmogenic right ventricular cardiomyopathy; PAWPM, mean pulmonary capillary wedge pressure as measured by RHC (PNG 40 KB) [file 392_2018_1399_MOESM1_ESM.png]
